# Update of Prior Probabilities by Minimal Divergence

**DOI:** 10.3390/e23121668

**Published:** 2021-12-11

**Authors:** Jan Naudts

**Affiliations:** Departement Fysica, Universiteit Antwerpen, 2610 Antwerpen, Belgium; Jan.Naudts@uantwerpen.be

**Keywords:** statistical update procedure, minimal divergence, Hellinger distance, Bregman divergence, Jeffrey conditioning

## Abstract

The present paper investigates the update of an empirical probability distribution with the results of a new set of observations. The update reproduces the new observations and interpolates using prior information. The optimal update is obtained by minimizing either the Hellinger distance or the quadratic Bregman divergence. The results obtained by the two methods differ. Updates with information about conditional probabilities are considered as well.

## 1. Introduction

The present work is inspired by the current practices in Information Geometry [1,2,3] where minimization of divergences is an important tool. In Statistical Physics a divergence is called a relative entropy. Its importance was noted rather late in the twentieth century, after the work of Jaynes on the maximal entropy principle [4]. Estimation in the presence of hidden variables by minimizing a divergence function is briefly discussed in Chapter 8 of [2].

Assume now that some observation or experiment yields new statistical data. The approach is then to look for a probability distribution that reproduces the newly observed probabilities and that interpolates the data with missing information coming from a prior.

No further model assumptions are imposed. Hence, the statistical model under consideration consists of all probability distributions that are consistent with the newly obtained empirical data. Internal consistency of the empirical data ensures that the model is not empty. The update is the model point that minimizes the chosen divergence function from the prior to the manifold of the model.

In the context of Maximum Likelihood Estimation (MLE) one usually adopts a parameterized model. The dimension of the model can be kept low and properties of the model can be used to ease the calculations. One assumes that the new data can lead to a more accurate estimation of the limited number of model parameters. It can then happen that the model is misspecified [5] and that the update is only a good approximation of the empirical data.

Here, the model is dictated by the newly acquired empirical data and the update is forced to reproduce the measured data. Finding the probability distribution is then an underdetermined problem. Minimization of the divergence from the prior probability distribution solves the underdetermination.

In Bayesian statistics, the update q(B) of the probability p(B) of an event *B* equals

(1) $$\begin{array}{ccc} q(B) & = & p^{\mathrm{emp}}\left(A\right)\;p\left(B|A\right)+p^{\mathrm{emp}}\left(A^{c}\right)\;p\left(B|A^{c}\right). \end{array}$$

The quantities $p^{\mathrm{emp}}\left(A\right)$ and $p^{\mathrm{emp}}\left(A^{c}\right)$ are the empirical probabilities obtained after repeated measurement of event *A* and its complement $A^{c}$. Expression (1) has been called *Jeffrey conditioning* [6]. It implies the sufficiency conditions q(B|A)=p(B|A) and $q\left(B|A^{c}\right)=p\left(B|A^{c}\right)$. It is an updating rule used in Radical Probabilism [7]. This expression is also obtained when minimizing the Hellinger distance between the prior and the model manifold. A proof of the latter follows later on in Section 4.

The present approach is a special case of minimizing a divergence function in the presence of linear constraints. See the introduction of [8] for an overview of early applications of this technique. Two classes of generalized distance functions satisfy a natural set of axioms: the f-divergences of Csiszár and the generalized Bregman divergences. The squared Hellinger distance belongs to the former class. The other divergence function considered here is the square Bregman divergence. Both Hellinger and square Bregman have special properties that make it easy to work with them.

A broad class of generalized Bregman divergences satisfies the Pythagorean equality [8,9]. Pythagorean inequalities hold for an even larger class [10]. The Pythagorean relations derived in the present work make use of the specific properties of the Hellinger distance and of the quadratic Bregman divergence. It is unclear how to prove them for more general divergences.

One incentive for starting the present work is a paper of Banerjee, Guo, and Wang [11,12]. They consider the problem of predicting a random variable $Z_{1}$ given observations of a random variable $Z_{2}$. It is well-known that the conditional expectation, as defined by Kolmogorov, is the optimal predictor. They show that this statement remains true when the metric distance is replaced by a Bregman divergence. It is shown in Theorem 2 below that a proof in a more general context yields a deviating result.

The next Section fixes notations. Section 3 collects some results about the squared Hellinger distance and the quadratic Bregman divergence. Section 4 discusses the optimal choice and contains the Theorems 1 and 2. The proof of the theorems can be adapted to cover the situation that a subsequent measurement also yields information on conditional probabilities. This is shown in Section 4.3. Section 5 treats a simple example. A final section summarizes the results of the paper.

## 2. Empirical Data

Consider a probability space Ω,μ. A measurable subset *A* of Ω is called an event. Its probability is denoted p(A) and is given by

$$\begin{array}{ccc} p(A) & = & \int_{\Omega }I_{A}\left(x\right)\;d\mu \left(x\right), \end{array}$$

where $I_{A}\left(x\right)$ equals 1 when x∈A and 0 otherwise. The conditional expectation of a random variable *f* given an event *A* with non-vanishing probability p(A) is given by

$$\begin{array}{ccc} E_{\mu }f|A & = & \frac{1}{p(A)}E_{\mu }f\;I_{A}. \end{array}$$

The probability space Ω,μ reflects the prior knowledge of the system at hand. When new data become available an update procedure is used to select the posterior probability space. The latter is denoted Ω,ν in what follows. The corresponding probability of an event *A* is denoted q(A).

The outcome of repeated experiments is the empirical probability distribution of the events, denoted $p^{\mathrm{emp}}\left(A\right)$. The question at hand is then to establish a criterion for finding the update ν of the probability distribution μ that is as close as possible to μ while reproducing the empirical results.

The event *A* defines a partition $A,A^{c}$ of the probability space Ω,μ. As before $A^{c}$ denotes the complement of *A* in Ω. In what follows a slightly more general situation is considered in which the event *A* is replaced by a partition ${\left(O_{i}\right)}_{i=1}^{n}$ of the measure space Ω,μ into subsets with non-vanishing probability. The notations $p_{i}$ and $\mu_{i}$ are used, with

(2) $$\begin{array}{c} p_{i}=p\left(O_{i}\right)\;\mathrm{and}\;\;d\mu_{i}\left(x\right)=\frac{1}{p_{i}}I_{O_{i}}\left(x\right)\;d\mu \left(x\right). \end{array}$$

Introduce the random variable *g* defined by g(x)=i when $x\in O_{i}$. Repeated measurement of the random variable *g* yields the empirical probabilities

$$\begin{array}{ccc} p_{i}^{\mathrm{emp}} & = & \mathrm{Emp}\;\mathrm{Prob}\{x:\;g(x)=i\}. \end{array}$$

They may deviate from the prior probabilities $p_{i}$. In some cases one also measures the conditional probabilities

$$\begin{array}{ccc} p^{\mathrm{emp}}\left(B|O_{i}\right) & = & \mathrm{Emp}\;\mathrm{Prob}\;\mathrm{of}B\mathrm{given}\;\mathrm{that}g(x)=i \end{array}$$

of some other event *B*.

## 3. A Geometric Approach

In this section two divergences are reviewed, the squared Hellinger distance and the quadratic Bregman divergence.

### 3.1. Squared Hellinger Distance

For simplicity the present section is restricted to the case that the sample space Ω is the real line.

Given two probability measures μ and σ, both absolutely continuous w.r.t. the Lebesgue measure, the squared Hellinger distance is the divergence $D_{2}\left(\sigma ||\mu \right)$ defined by

$$\begin{array}{ccc} D_{2}\left(\sigma ||\mu \right) & = & \frac{1}{2}\int_{R}{\left(\sqrt{\frac{\;d\sigma }{\;dx}}-\sqrt{\frac{\;d\mu }{\;dx}}\right)}^{2}\;dx. \end{array}$$

It satisfies

$$\begin{array}{ccc} D_{2}\left(\sigma ||\mu \right) & = & 1-\int_{R}\sqrt{\frac{\;d\sigma }{\;dx}\frac{\;d\mu }{\;dx}}\;dx. \end{array}$$

Let ${\left(O_{i}\right)}_{i}$ be a partition of Ω,μ and let g(x)=i when *x* belongs to $O_{i}$, as before. Let $p_{i}$ and $\mu_{i}$ be defined by (2). Consider the following functions of *i*, with *i* in {1,…,n},

$$\begin{array}{ccc} \tau^{\left(1\right)}\left(i\right) & = & \mu ,\;\mathrm{independent}\;\mathrm{of}i,\\ \tau^{\left(2\right)}\left(i\right) & = & \mu_{i},\\ \tau^{\left(3\right)}\left(i\right) & = & \sigma_{i}, \end{array}$$

where each of the $\sigma_{i}$ is a probability distribution with support in $O_{i}$. The empirical expectation of a function f(i) is given by $E^{\mathrm{emp}}f=\sum_{i}p_{i}^{\mathrm{emp}}f\left(i\right)$.

**Proposition** **1.** *If $p_{i}^{\mathrm{emp}}>0$ for all i and $\sum_{i}p_{i}^{\mathrm{emp}}=1$ then one has*

$$\begin{array}{c} E^{\mathrm{emp}}D_{2}(\tau^{\left(1\right)}\left|\right|\tau^{\left(3\right)})\geq E^{\mathrm{emp}}D_{2}(\tau^{\left(1\right)}\left|\right|\tau^{\left(2\right)}) \end{array}$$

*with equality if and only if $\sigma_{i}=\mu_{i}$ for all i.*

First prove the following two lemmas.

**Lemma** **1.** *Assume that the probability measure $\nu_{i}$ is absolutely continuous w.r.t. the measure $\mu_{i}$, with Radon-Nikodym derivative given by $\;d\nu_{i}\left(x\right)=f_{i}\left(x\right)\;d\mu_{i}$. Then one has*

$$\begin{array}{ccc} D_{2}\left(\mu |\right|\sigma_{i})-D_{2}\left(\mu |\right|\nu_{i}) & = & \sqrt{p_{i}}\left[D_{2}(\mu_{i}\left|\right|\sigma_{i})-D_{2}(\mu_{i}\left|\right|\nu_{i})\right] \end{array}$$

*and*

$$\begin{array}{ccc} D_{2}(\mu_{i}\left|\right|\nu_{i}) & = & 1-\int_{O_{i}}\sqrt{f_{i}\left(x\right)}\;d\mu_{i}\left(x\right). \end{array}$$

**Proof.**  One calculates

$$\begin{array}{ccc} D_{2}\left(\mu |\right|\sigma_{i})-D_{2}\left(\mu |\right|\nu_{i}) & = & \int_{R}\sqrt{\frac{\;d\mu }{\;dx}}\left[\sqrt{\frac{\;d\nu_{i}}{\;dx}}-\sqrt{\frac{\;d\sigma_{i}}{\;dx}}\right]\;dx\\ & = & \sqrt{p_{i}}\int_{O_{i}}\sqrt{\frac{\;d\mu_{i}}{\;dx}}\left[\sqrt{\frac{\;d\nu_{i}}{\;dx}}-\sqrt{\frac{\;d\sigma_{i}}{\;dx}}\right]\;dx\\ & = & \sqrt{p_{i}}\left[\int_{O_{i}}\sqrt{f_{i}\left(x\right)}\;d\mu_{i}\left(x\right)-\int_{O_{i}}{\left[\frac{\;d\mu_{i}}{\;dx}\frac{\;d\sigma_{i}}{\;dx}\right]}^{1/2}\;dx\right]\\ & = & \sqrt{p_{i}}\left[\int_{O_{i}}\sqrt{f_{i}\left(x\right)}\;d\mu_{i}\left(x\right)-1+D_{2}(\mu_{i}\left|\right|\sigma_{i})\right]. \end{array}$$

Now take $\sigma_{i}=\nu_{i}$ to obtain the desired results. □

**Lemma** **2.** *(Pythagorean relation) For any i is*

$$\begin{array}{ccc} D_{2}\left(\mu |\right|\sigma_{i}) & = & D_{2}\left(\mu |\right|\mu_{i})+\sqrt{p_{i}}D_{2}(\mu_{i}\left|\right|\sigma_{i}). \end{array}$$

**Proof.**  The proof follows by taking $\nu_{i}=\mu_{i}$ in the previous lemma. □

**Proof.**  (Proposition 1)

From the previous lemma it follows that $D_{2}(\tau^{\left(1\right)}\left|\right|\tau^{\left(3\right)})\geq D_{2}(\tau^{\left(1\right)}\left|\right|\tau^{\left(2\right)})$. Note that $\sigma_{i}=\mu_{i}$ implies that $\tau^{\left(3\right)}=\tau^{\left(2\right)}$ and hence $D_{2}(\tau^{\left(1\right)}\left|\right|\tau^{\left(3\right)})=D_{2}(\tau^{\left(1\right)}\left|\right|\tau^{\left(2\right)})$. Conversely, if

$$\begin{array}{ccc} E^{\mathrm{emp}}D_{2}(\tau^{\left(1\right)}\left|\right|\tau^{\left(3\right)}) & = & E^{\mathrm{emp}}D_{2}(\tau^{\left(1\right)}\left|\right|\tau^{\left(2\right)}) \end{array}$$

then it follows from the previous lemma that $E^{\mathrm{emp}}D_{2}(\tau^{\left(2\right)}\left|\right|\tau^{\left(3\right)})=0$. If in addition $p_{i}^{\mathrm{emp}}>0$ for all *i* then it follows that for all *i*

$$\begin{array}{ccc} 0 & = & D_{2}\left(\tau^{\left(2\right)}\left(i\right)\left|\right|\tau^{\left(3\right)}\left(i\right)\right). \end{array}$$

Because the squared Hellinger distance is a divergence, this implies that $\tau^{\left(2\right)}\left(i\right)=\tau^{\left(3\right)}\left(i\right)$, which is equivalent with $\mu_{i}=\sigma_{i}$. □

### 3.2. Bregman Divergence

In the present section the squared Hellinger distance, which is an f-divergence, is replaced by a divergence of the Bregman type. In addition let Ω be a finite set equipped with the counting measure ρ. It assigns to each subset *A* of Ω the number of elements in *A*. This number is denoted |A|. The expectation value $E_{\mu }f$ of a random variable *f* w.r.t. the probability measure μ is given by

$$\begin{array}{ccc} E_{\mu }f & = & \sum_{k\in \Omega }\mu \left(k\right)f\left(k\right). \end{array}$$

Given a partition of Ω into sets $O_{i}$ one can define conditional probability measures with probability mass function $\rho_{i}$ given by

(3) $$\begin{array}{ccc} \rho_{i}\left(k\right) & = & \frac{1}{|O_{i}|}\;\mathrm{if}\;k\in O_{i},\\ & = & 0\;\mathrm{otherwise}. \end{array}$$

Similarly, conditional probability measures with probability mass function $\mu_{i}$ are given by

(4) $$\begin{array}{ccc} \mu_{i}\left(k\right) & = & \frac{\mu (k)}{\mu (O_{i})}\;\mathrm{if}k\in O_{i},\\ & = & 0\;\mathrm{otherwise}. \end{array}$$

Fix a strictly convex function ϕ:R↦R. The Bregman divergence of the probability measures σ and μ is defined by

$$\begin{array}{ccc} D_{\phi }\left(\sigma ||\mu \right) & = & F(\sigma )-F(\mu )-\langle \nabla F,\sigma -\mu \rangle \end{array}$$

with

$$\begin{array}{c} F\left(\sigma \right)=\sum_{k}\phi \left(\sigma (k)\right)\;\mathrm{and}\;\nabla_{k}F\left(\sigma \right)=\phi^{\prime }\left(\sigma (k)\right). \end{array}$$

In the case that $\phi \left(x\right)=x^{2}/2$, which is used below, it becomes

(5) $$\begin{array}{ccc} D_{\phi }\left(\sigma ||\mu \right) & = & \frac{1}{2}\sum_{k}{\left[\sigma (k)-\mu (k)\right]}^{2}. \end{array}$$

For convenience, this case is referred to as the *quadratic Bregman divergence*.

The following result, obtained with the quadratic Bregman divergence, is more elegant than the result of Lemma 2.

**Proposition** **2.** *Consider the quadratic Bregman divergence $D_{\phi }$ as given by (5). Let $\nu_{i}=p_{i}\mu_{i}+\left(1-p_{i}\right)\rho_{i}$. Let $\sigma_{i}$ be any probability measure with support in $O_{i}$. Then the following Pythagorean relation holds.*

$$\begin{array}{ccc} D_{\phi }\left(\mu |\right|\sigma_{i}) & = & D_{\phi }\left(\mu |\right|\nu_{i})+D_{\phi }(\nu_{i}\left|\right|\sigma_{i}). \end{array}$$

**Proof.**  One calculates

$$\begin{array}{ccc} D_{\phi }\left(\mu |\right|\sigma_{i})-D_{\phi }\left(\mu |\right|\nu_{i}) & = & D_{\phi }(\nu_{i}\left|\right|\sigma_{i})+\sum_{x}\left[\mu \left(x\right)-\nu_{i}\left(x\right)\right]\;\left[\phi^{\prime }\left(\nu_{i}\left(x\right)\right)-\phi^{\prime }\left(\sigma_{i}\left(x\right)\right)\right]\\ & = & D_{\phi }(\nu_{i}\left|\right|\sigma_{i})+\sum_{x\in O_{i}}\left[p_{i}\mu_{i}\left(x\right)-\nu_{i}\left(x\right)\right]\;\left[\phi^{\prime }\left(\nu_{i}\left(x\right)\right)-\phi^{\prime }\left(\sigma_{i}\left(x\right)\right)\right]\\ & = & D_{\phi }(\nu_{i}\left|\right|\sigma_{i})-\left(1-p_{i}\right)\frac{1}{|O_{i}|}\sum_{x\in O_{i}}\left[\phi^{\prime }\left(\nu_{i}\left(x\right)\right)-\phi^{\prime }\left(\sigma_{i}\left(x\right)\right)\right]. \end{array}$$

Use now that $\phi^{\prime }\left(u\right)=u$ and the normalization of the probability measures $\nu_{i}$ and $\sigma_{i}$ to find the desired result. □

## 4. The Optimal Choice

### 4.1. Updated Probabilities

The following result proves that the standard Kolmogorovian definition of the conditional probability minimizes the Hellinger distance between the prior probability measure μ and the updated probability measure ν. The optimal choice of the updated probability measure ν is given by corresponding probabilities q(B). They satisfy

$$\begin{array}{ccc} q(B) & = & \sum_{i=1}^{n}p_{i}^{\mathrm{emp}}p\left(B|O_{i}\right)\;\mathrm{for}\;\mathrm{any}\;\mathrm{event}B. \end{array}$$

**Theorem** **1.** *Let be given a partition ${\left(O_{i}\right)}_{i=1}^{n}$ of the probability space Ω,μ with Ω=R. Let $\mu_{i}$ be given by (2). Let $p_{i}=p\left(O_{i}\right)>0$ denote the probability of the event $O_{i}$ and let be given strictly positive empirical probabilities $p_{i}^{\mathrm{emp}}$, i=1,…,n. The squared Hellinger distance $D_{2}\left(\sigma ||\mu \right)$ as a function of σ is minimal if and only if $\sigma_{i}=\mu_{i}$ for all i. Here, σ is any probability measure on *Ω* satisfying*

$$\begin{array}{c} \sigma =\sum_{i=1}^{n}p_{i}^{\mathrm{emp}}\sigma_{i}, \end{array}$$

*and each of the $\sigma_{i}$ is a probability measure with support in $O_{i}$ and absolutely continuous w.r.t. $\mu_{i}$.*

Note that the probability measure ν given by

$$\begin{array}{ccc} \nu (x) & = & \sum_{i=1}^{n}p_{i}^{\mathrm{emp}}\mu_{i}\left(x\right) \end{array}$$

uses the Kolmogorovian conditional probability as the predictor because the probabilities determined by the $\mu_{i}$ are obtained from the prior probability distribution μ by $p_{i}\left(x\right)=p\left(x|O_{i}\right)$. By the above theorem this predictor is the optimal one w.r.t. the squared Hellinger distance.

**Proof.**  With the notations of the previous section is

$$\begin{array}{ccc} D_{2}\left(\sigma ||\mu \right) & = & E^{\mathrm{emp}}D_{2}(\tau^{\left(1\right)}\left|\right|\tau^{\left(3\right)}). \end{array}$$

Proposition 1 shows that it is minimal if and only if $\sigma_{i}=\mu_{i}$ for all *i*. □

Next, consider the use of the quadratic Bregman divergence in the context of a finite probability space.

**Theorem** **2.** *Let be given a partition ${\left(O_{i}\right)}_{i=1}^{n}$ of the finite probability space Ω,μ. Let $\rho_{i}$ be the counting measure on $O_{i}$ defined by (3). Let $\mu_{i}$ be given by (2). Let $p_{i}=p\left(O_{i}\right)>0$ denote the probability of the event $O_{i}$ and let be given strictly positive empirical probabilities $p_{i}^{\mathrm{emp}}$, i=1,…,n summing up to 1. Assume that*

(6) $$\begin{array}{c} p_{i}^{\mathrm{emp}}\geq p_{i}\left[1-|O_{i}|\mu_{i}\left(x\right)\right]\;for\;all\;x\in O_{i}\;and\;for\;i=1,\ldots ,n. \end{array}$$

*Then the following hold.*

(*a*) *A probability distribution ν is defined by $\nu =\sum_{i}p_{i}^{\mathrm{emp}}\nu_{i}$ with*
(7) $$\begin{array}{ccc} \nu_{i} & = & \left(1-\frac{p_{i}}{p_{i}^{\mathrm{emp}}}\right)\rho_{i}+\frac{p_{i}}{p_{i}^{\mathrm{emp}}}\mu_{i}. \end{array}$$
(*b*) *Let σ be any probability measure on Ω satisfying $\sigma =\sum_{i=1}^{n}p_{i}^{\mathrm{emp}}\sigma_{i}$, where each of the $\sigma_{i}$ is a probability distribution with support in $O_{i}$. Then the quadratic Bregman divergence satisfies the Pythagorean relation*
(8) $$\begin{array}{ccc} D_{\phi }\left(\sigma ||\mu \right) & = & D_{\phi }\left(\nu ||\mu \right)+\sum_{i=1}^{n}{\left(p_{i}^{\mathrm{emp}}\right)}^{2}D_{\phi }(\sigma_{i}\left|\right|\nu_{i}). \end{array}$$
(*c*) *The quadratic Bregman divergence $D_{\phi }\left(\sigma ||\mu \right)$ is minimal if and only if σ=ν.*

**Proof.** 

(a)

The assumption (6) guarantees that the $\nu_{i}\left(x\right)$ are probabilities.

(b)

One calculates

$$\begin{array}{ccc} D_{\phi }\left(\sigma ||\mu \right)-D_{\phi }\left(\nu ||\mu \right) & = & \frac{1}{2}\sum_{x}\left[\sigma (x)-\nu (x)\right]\;\left[\sigma (x)+\nu (x)-2\mu (x)\right]\\ & = & \sum_{i=1}^{n}p_{i}^{\mathrm{emp}}\frac{1}{2}\sum_{x\in O_{i}}\left[\sigma_{i}\left(x\right)-\nu_{i}\left(x\right)\right]\\ & & \times \left[p_{i}^{\mathrm{emp}}\sigma_{i}\left(x\right)+p_{i}^{\mathrm{emp}}\nu_{i}\left(x\right)-2p_{i}\mu_{i}\left(x\right)\right]\\ & = & \sum_{i=1}^{n}{\left(p_{i}^{\mathrm{emp}}\right)}^{2}\frac{1}{2}\sum_{x\in O_{i}}{\left[\sigma_{i}\left(x\right)-\nu_{i}\left(x\right)\right]}^{2}\\ & & +\sum_{i=1}^{n}p_{i}^{\mathrm{emp}}\sum_{x\in O_{i}}\left[\sigma_{i}\left(x\right)-\nu_{i}\left(x\right)\right]\left(p_{i}^{\mathrm{emp}}-p_{i}\right)\rho_{i}\left(x\right)\\ & = & \sum_{i=1}^{n}{\left(p_{i}^{\mathrm{emp}}\right)}^{2}D_{\phi }(\sigma_{i}\left|\right|\nu_{i}). \end{array}$$

In the above calculation the third line is obtained by eliminating $p_{i}\mu_{i}$ using the definition of $\nu_{i}$. This gives

$$\begin{array}{ccc} & & p_{i}^{\mathrm{emp}}\sigma_{i}\left(x\right)+p_{i}^{\mathrm{emp}}\nu_{i}\left(x\right)-2p_{i}\mu_{i}\left(x\right)\\ & = & p_{i}^{\mathrm{emp}}\sigma_{i}\left(x\right)+p_{i}^{\mathrm{emp}}\nu_{i}\left(x\right)-2p_{i}^{\mathrm{emp}}\left[\nu_{i}\left(x\right)-\left(1-\frac{p_{i}}{p_{i}^{\mathrm{emp}}}\right)\rho_{i}\left(x\right)\right]\\ & = & p_{i}^{\mathrm{emp}}\left[\sigma_{i}\left(x\right)-\nu_{i}\left(x\right)\right]+2\left(p_{i}^{\mathrm{emp}}-p_{i}\right)\rho_{i}\left(x\right). \end{array}$$

The term

$$\begin{array}{c} \sum_{i=1}^{n}p_{i}^{\mathrm{emp}}\sum_{x\in O_{i}}\left[\sigma_{i}\left(x\right)-\nu_{i}\left(x\right)\right]\left(p_{i}^{\mathrm{emp}}-p_{i}\right)\rho_{i}\left(x\right) \end{array}$$

vanishes because $\rho_{i}\left(x\right)$ is constant on the set $O_{i}$ and the probability measures $\nu_{i}$ and $\sigma_{i}$ have support in $O_{i}$.

(c)

From (b) it follows that $D_{\phi }\left(\sigma ||\mu \right)\geq D_{\phi }\left(\nu ||\mu \right)$, with equality when σ=ν.

Conversely, when $D_{\phi }\left(\sigma ||\mu \right)=D_{\phi }\left(\nu ||\mu \right)$ then (8) implies that

$$\begin{array}{ccc} \sum_{i=1}^{n}{\left(p_{i}^{\mathrm{emp}}\right)}^{2}D_{\phi }(\sigma_{i}\left|\right|\nu_{i}) & = & 0. \end{array}$$

The empirical probabilities are strictly positive by assumption. Hence, it follows that $D_{\phi }\left(\mu |\right|\sigma_{i})=D_{\phi }\left(\mu |\right|\nu_{i})$ for all *i* and hence, that $\sigma_{i}=\nu_{i}$ for all *i*. The latter implies σ=ν. □

The optimal update ν can be written as

$$\begin{array}{c} \nu =\sum_{i}\left[\left(p_{i}^{\mathrm{emp}}-p_{i}\right)\rho_{i}+p_{i}\mu_{i}\right]=\mu +\sum_{i}\left(p_{i}^{\mathrm{emp}}-p_{i}\right)\rho_{i}. \end{array}$$

This result is in general quite different from the update proposed by Theorem 1, which is

$$\begin{array}{ccc} \nu & = & \sum_{i}p_{i}^{\mathrm{emp}}\mu_{i}. \end{array}$$

The updates proposed by the two theorems coincide only in the special cases that either $p_{i}^{\mathrm{emp}}=p_{i}$ for all *i* or that $\mu_{i}=\rho_{i}$ for all *i*. In the latter case the prior distribution $\mu =\sum_{i}p_{i}\rho_{i}$ is replaced by the update $\nu =\sum_{i}p_{i}^{\mathrm{emp}}\rho_{i}$.

The entropy of the update when event $O_{i}$ is observed, according to Theorem 1, equals $S\left(\nu_{i}\right)=S\left(\mu_{i}\right)$. According to Theorem 2 it equals

$$\begin{array}{ccc} S(\nu_{i}) & = & S\left(\left[1-\frac{p_{i}}{p_{i}^{\mathrm{emp}}}\right]\rho_{i}+\frac{p_{i}}{p_{i}^{\mathrm{emp}}}\mu_{i}\right). \end{array}$$

If $p_{i}\leq p_{i}^{\mathrm{emp}}$ then it follows that

$$\begin{array}{ccc} S(\nu_{i}) & \geq & \left[1-\frac{p_{i}}{p_{i}^{\mathrm{emp}}}\right]S\left(\rho_{i}\right)+\frac{p_{i}}{p_{i}^{\mathrm{emp}}}S\left(\mu_{i}\right)\\ & \geq & S(\mu_{i}). \end{array}$$

The former inequality follows because the entropy is a concave function. The latter follows because entropy is maximal for the uniform distribution $\rho_{i}$. On the other hand, if $p_{i}>p_{i}^{\mathrm{emp}}$ then one has

$$\begin{array}{ccc} S(\mu_{i}) & = & S\left(\left[1-\frac{p_{i}^{\mathrm{emp}}}{p_{i}}\right]\rho_{i}+\frac{p_{i}^{\mathrm{emp}}}{p_{i}}\nu_{i}\right)\\ & \geq & \left[1-\frac{p_{i}^{\mathrm{emp}}}{p_{i}}\right]S\left(\rho_{i}\right)+\frac{p_{i}^{\mathrm{emp}}}{p_{i}}S\left(\nu_{i}\right)\\ & \geq & S(\nu_{i}). \end{array}$$

In the latter case the decrease of the entropy is stronger than in the case of the update based on the squared Hellinger distance. In conclusion, the update relying on the quadratic Bregman divergence looses details of the prior distribution by making a convex combination with a uniform distribution weighed with the probabilities of the observation. It does this moreso for the events with observed probability larger than predicted; this is when $p_{i}^{\mathrm{emp}}>p_{i}$.

Note that Theorem 2 cannot always be applied because it contains restrictions on the empirical probabilities. In particular, if the prior probability μ(x) of some point *x* in Ω vanishes then the condition (6) requires that the empirical probability $p_{i}^{\mathrm{emp}}$ of the partition $O_{i}$ to which the point *x* belongs is larger than or equal to the prior probability $p_{i}$.

### 4.2. Update of Conditional Probabilities

The two previous theorems assume that no empirical information is available about conditional probabilities. If such information is present then an optimal choice should make use of it. In one case the solution of the problem is straightforward. If the probabilities $p_{i}^{\mathrm{emp}}$ are available together with all conditional probabilities $p^{\mathrm{emp}}\left(B|O_{i}\right)$ and there exists an update ν which reproduces these results then it is unique. Two cases remain: (1) The information about the conditional probabilities is incomplete; (2) the information is internally inconsistent – no update exists which reproduces the data.

Let us tackle the problem by considering the case that the only information that is available besides the probabilities $p_{i}^{\mathrm{emp}}$ is the vector of conditional probabilities $p^{\mathrm{emp}}\left(B|O_{i}\right)$ of a fixed event *B*, given the outcome of the measurement of the random variable *g* as introduced in Section 2.

The following result is independent of the choice of divergence function.

**Proposition** **3.** *Fix an event B in Ω. Assume that the conditional probabilities $p(B|O_{i})$, i=1,…,n, are strictly positive and strictly less than 1. Assume in addition that $p_{i}^{\mathrm{emp}}p^{\mathrm{emp}}\left(B|O_{i}\right)\leq 1$ for all i. Then there exists an update ν with corresponding probabilities q(·) such that $q\left(O_{i}\right)=p_{i}^{\mathrm{emp}}$ and $q\left(B|O_{i}\right)=p^{\mathrm{emp}}\left(B|O_{i}\right)$, i=1,…,n.*

**Proof.**  An obvious choice is to take ν of the form $\nu =\sum_{i}p_{i}^{\mathrm{emp}}\nu_{i}$ with $\nu_{i}$ of the form

$$\begin{array}{c} \;d\nu_{i}\left(x\right)=\left[a_{i}I_{B\cap O_{i}}\left(x\right)+b_{i}I_{B^{c}\cap O_{i}}\left(x\right)\right]\;d\mu \left(x\right), \end{array}$$

with $a_{i}\geq 0$ and $b_{i}\geq 0$. Normalization of the $\nu_{i}$ gives the conditions

(9) $$\begin{array}{ccc} 1 & = & a_{i}p\left(B\cap O_{i}\right)+b_{i}p\left(B^{c}\cap O_{i}\right). \end{array}$$

Reproduction of the conditional probabilities gives the conditions

$$\begin{array}{c} p^{\mathrm{emp}}\left(B|O_{i}\right)=\frac{q(B\cap O_{i})}{q(O_{i})}=a_{i}\frac{p(B\cap O_{i})}{p_{i}^{\mathrm{emp}}}. \end{array}$$

The latter gives

$$\begin{array}{c} a_{i}=\frac{p_{i}^{\mathrm{emp}}}{p_{i}}\;\frac{p^{\mathrm{emp}}\left(B|O_{i}\right)}{p(B|O_{i})}. \end{array}$$

The normalization condition (9) becomes

$$\begin{array}{c} 1=p_{i}^{\mathrm{emp}}p^{\mathrm{emp}}\left(B|O_{i}\right)+b_{i}p\left(B^{c}\cap O_{i}\right). \end{array}$$

It has a positive solution for $b_{i}$ because $p_{i}^{\mathrm{emp}}p^{\mathrm{emp}}\left(B|O_{i}\right)\leq 1$ and $p(B^{c}\cap O_{i})>0$. □

### 4.3. The Hellinger Case

The optimal updates can be derived easily from Theorem 1. Double the partition by introduction of the following sets

$$\begin{array}{c} O_{i}^{+}=B\cap O_{i}\;and\;O_{i}^{-}=B^{c}\cap O_{i}. \end{array}$$

They have prior probabilities $p_{i}^{\pm }=p\left(O_{i}^{\pm }\right)$. Corresponding prior measures $\mu_{i}^{\pm }$ are defined by

$$\begin{array}{c} \;d\mu_{i}^{\pm }\left(x\right)=\frac{1}{p_{i}^{\pm }}I_{O_{i}^{\pm }}\left(x\right)\;d\mu \left(x\right). \end{array}$$

The empirical probability of the set $O_{i}^{+}$ is taken equal to $p_{i}^{\mathrm{emp}}p^{\mathrm{emp}}\left(B|O_{i}\right)$, that of $O_{i}^{-}$ equals $p_{i}^{\mathrm{emp}}\left[1-p^{\mathrm{emp}}\left(B|O_{i}\right)\right]$. The optimal update ν follows from Theorem 1 and is given by

$$\begin{array}{ccc} \;d\nu (x) & = & \sum_{i}p_{i}^{\mathrm{emp}}p^{\mathrm{emp}}\left(B|O_{i}\right)\;\;d\mu_{i}^{+}\left(x\right)+\sum_{i}p_{i}^{\mathrm{emp}}\left[1-p^{\mathrm{emp}}\left(B|O_{i}\right)\right]\;\;d\mu_{i}^{-}\left(x\right). \end{array}$$

By construction it is

$$\begin{array}{c} q\left(O_{i}^{+}\right)=p_{i}^{\mathrm{emp}}p^{\mathrm{emp}}\left(B|O_{i}\right)\;and\;q\left(O_{i}^{-}\right)=p_{i}^{\mathrm{emp}}\left[1-p^{\mathrm{emp}}\left(B|O_{i}\right)\right]. \end{array}$$

One now verifies that $q\left(O_{i}\right)=p_{i}^{\mathrm{emp}}$ and $q\left(B|O_{i}\right)=p^{\mathrm{emp}}\left(B|O_{i}\right)$, which is the intended result.

### 4.4. The Bregman Case

Next consider the optimization with the quadratic Bregman divergence. Probability distributions $\rho_{i}^{\pm }$ are defined by

$$\begin{array}{ccc} \rho_{i}^{\pm }\left(x\right) & = & \frac{1}{|O_{i}^{\pm }|}I_{O_{i}^{\pm }}\left(x\right). \end{array}$$

Introduce the notations

$$\begin{array}{ccc} r_{i}^{+} & = & \frac{p_{i}^{+}}{p_{i}^{\mathrm{emp}}p^{\mathrm{emp}}\left(B|O_{i}\right)},\\ r_{i}^{-} & = & \frac{p_{i}^{-}}{p_{i}^{\mathrm{emp}}\left[1-p^{\mathrm{emp}}\left(B|O_{i}\right)\right]},\\ \nu_{i}^{\pm }\left(x\right) & = & \left(1-r_{i}^{\pm }\right)\rho_{i}^{\pm }+r_{i}^{\pm }\mu_{i}^{\pm }\left(x\right). \end{array}$$

Then the condition for Theorem 2 to hold is that $\nu_{i}^{\pm }\left(x\right)\geq 0$ for all x,i. The optimal probability distribution ν is given by

$$\begin{array}{ccc} \nu (x) & = & \sum_{i}p_{i}^{\mathrm{emp}}p^{\mathrm{emp}}\left(B|O_{i}\right)\nu_{i}^{+}\left(x\right)+\sum_{i}p_{i}^{\mathrm{emp}}\left[1-p^{\mathrm{emp}}\left(B|O_{i}\right)\right]\nu_{i}^{-}\left(x\right)\\ & = & \sum_{i}\left[p_{i}^{\mathrm{emp}}p^{\mathrm{emp}}\left(B|O_{i}\right)-p_{i}^{+}\right]\rho_{i}^{+}+\sum_{i}p_{i}^{+}\mu_{i}^{+}\\ & & +\sum_{i}\left[p_{i}^{\mathrm{emp}}\left[1-p^{\mathrm{emp}}\left(B|O_{i}\right)\right]-p_{i}^{-}\right]\rho_{i}^{-}+\sum_{i}p_{i}^{-}\mu_{i}^{-}\\ & = & \sum_{i}p_{i}^{\mathrm{emp}}p^{\mathrm{emp}}\left(B|O_{i}\right)\;\left[\rho_{i}^{+}-\rho_{i}^{-}\right]\\ & & -\sum_{i}p_{i}^{+}\rho_{i}^{+}+\sum_{i}\left[p_{i}^{\mathrm{emp}}-p_{i}^{-}\right]\rho_{i}^{-}\\ & & +\mu . \end{array}$$

## 5. Example

Assume that the prior probability distribution is binomial with parameters n,λ, where *n* is known with certainty. The probability mass function is given by

μ(k)=Prob(X=k)=nkλk(1−λ)n−kk=0,1,2,…,n.

The probability distribution and the value of the parameter λ are for instance the result of theoretical modeling of the experiment. Or they are obtained from a different kind of experiment.

The experiment under consideration yields accurate values for the probability $p^{\mathrm{emp}}$ of the two events X=1 and X=2. The problem at hand is to predict by extrapolation the probability of the event X=k for other values of *k*. A fit of the data with a binomial distribution is likely to fail because two accurate data points are given to determine a single parameter λ. The binomial model can be misspecified.

The geometric approach followed in the present paper yields an update from the binomial distribution to another distribution, one which is reproducing the data. The update is conducted in an unbiased manner. Quite often one is tempted to replace the model, in the case of the binomial model, by a model with one extra free parameter.

Let us see what are the results of minimizing divergence functions. The probability space Ω is the set of integers 0,1,2,…,n equipped with the uniform measure. Choose events

$$\begin{array}{c} O_{1}=\left\{1\right\},\;O_{2}=\left\{2\right\},\;O_{3}=\Omega \setminus \left(O_{1}\cup O_{2}\right). \end{array}$$

This gives for $p_{i}:=\mathrm{Prob}\;\left(X\in O_{i}\right)$

$$\begin{array}{c} p_{1}=\mu \left(1\right)=n\;\lambda {\left(1-\lambda \right)}^{n-1},\;p_{2}=\mu \left(2\right)=\frac{1}{2}n\left(n-1\right)\;\lambda^{2}{\left(1-\lambda \right)}^{n-2},\;p_{3}=1-p_{2}-p_{3}. \end{array}$$

The optimal update according to Theorem 1, minimizing the Hellinger distance, is given by the probabilities

$$\begin{array}{ccc} \nu (B) & = & \sum_{i}p_{i}^{\mathrm{emp}}\mu \left(B|O_{i}\right). \end{array}$$

In particular, the probability mass function ν(k):=ν({k}) becomes

$$\begin{array}{ccc} \nu (1) & = & p_{1}^{\mathrm{emp}},\\ \nu (2) & = & p_{2}^{\mathrm{emp}},\\ \nu (k) & = & \frac{p_{3}^{\mathrm{emp}}}{p_{3}}\;\mu \left(k\right)\;otherwise. \end{array}$$

The optimal update according to Theorem 2, minimizing the quadratic Bregman divergence, is given by (7). The auxiliary measures $\mu_{i}$, $\rho_{i}$, and $\nu_{i}$ have probability mass functions given by

$$\begin{array}{c} \mu_{i}\left(k\right)=\rho_{i}\left(k\right)=\nu_{i}=\delta_{k,i}\;\mathrm{for}i=1,2, \end{array}$$

and

$$\begin{array}{ccc} \mu_{3}\left(k\right) & = & \left(1-\delta_{k,1}\right)\left(1-\delta_{k,2}\right)\;\frac{\mu (k)}{p_{3}},\\ \rho_{3}\left(k\right) & = & \left(1-\delta_{k,1}\right)\left(1-\delta_{k,2}\right)\;\frac{1}{n-2}\\ \nu_{3}\left(k\right) & = & \left(1-\delta_{k,1}\right)\left(1-\delta_{k,2}\right)\;\left[\left(1-\frac{p_{3}}{p_{3}^{\mathrm{emp}}}\right)\frac{1}{n-2}+\frac{\mu (k)}{p_{3}^{\mathrm{emp}}}\right]. \end{array}$$

The probability mass function ν(k):=ν({k}) becomes

$$\begin{array}{ccc} \nu (k) & = & p_{1}^{\mathrm{emp}}\nu_{1}\left(k\right)+p_{2}^{\mathrm{emp}}\nu_{2}\left(k\right)+p_{3}^{\mathrm{emp}}\nu_{3}\left(k\right)\\ & = & p_{1}^{\mathrm{emp}}\;\mathrm{if}k=1,\\ & = & p_{2}^{\mathrm{emp}}\;\mathrm{if}k=2,\\ & = & \frac{p_{3}^{\mathrm{emp}}-p_{3}}{n-2}+\mu \left(k\right)\;\mathrm{otherwise}. \end{array}$$

The condition (6) is the requirement that all ν(k) are non-negative. Because the probabilities μ(k) can become very small this essentially means that $p_{3}^{\mathrm{emp}}$ should be larger than $p_{3}$. The amount of probability missing in the empirical probabilities $p_{1}^{\mathrm{emp}}$ and $p_{2}^{\mathrm{emp}}$ is equally distributed over the remaining n−1 points of Ω. On the other hand, when minimizing the Hellinger distance the excess or shortage of probability is compensated by multiplying all remaining probabilities by a constant factor.

**Figure 1:**
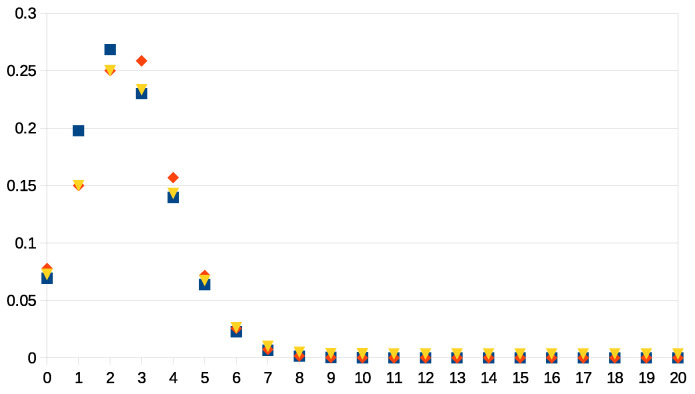
Probability as a function of the integer *k* running from 0 to 20, showing different updates of the binomial distribution with parameters n=20 and λ=1/8. The squares represent the binomial, the diamonds the update with the Hellinger distance, and the triangles the update with the square Bregman divergence. The empirical values are $p_{1}^{\mathrm{emp}}=0.15$ and $p_{2}^{\mathrm{emp}}=0.25$.

A numerical comparison with n=20 and λ=1/8 is found in Figure 1. The empirical values are $p_{1}^{\mathrm{emp}}=0.15$ and $p_{2}^{\mathrm{emp}}=0.25$. The difference with the prior values $p_{1}≃0.19774$ and $p_{2}≃0.26836$ is made large enough to amplify the effects of the update.

## 6. Summary

It is well known that the use of unmodified prior conditional probabilities is the optimal way for updating a probability distribution after new data become available. The update procedure minimizes the Hellinger distance between prior and posterior probability distributions. For the sake of completeness a proof is given in Theorem 1.

Alternatively, one can minimize the quadratic Bregman divergence instead of the Hellinger distance. The result is given in Theorem 2. The conservation of probability is handled in a different way in the two cases, either by multiplying prior probabilities with a suitable factor or by adding an appropriate term.

The example of Section 5 shows that the two update procedures have different effects and that neither of them may be satisfactory. This raises the question whether the present approach should be improved by choosing divergences other than Hellinger or Bregman.

In the present research, the work of Banerjee, Guo, and Wang [11] was considered as well. They prove that minimization of the Hellinger distance can be replaced by minimization of a Bregman divergence, without modifying the outcome. It is shown in Theorem 2 that, in a different context, the use of the Bregman divergence yields results quite distinct from those obtained by minimizing the Hellinger distance.

## References

[1] Amari S., Nagaoka H. (2000). Methods of Information Geometry.

[2] Amari S. (2016). Information Geometry and Its Applications.

[3] Ay N., Jost J., Lê H.V., Schwachhöfer L. (2017). Information Geometry.

[4] Jaynes E. (1957). Information theory and statistical mechanics. Phys. Rev..

[5] White H. (1982). Maximum Likelihood Estimation of Misspecified Models. Econometrica.

[6] Jeffrey R. (1987). Alias Smith and Jones: The Testimony of the Senses. Erkenntnis.

[7] Skyrms B. (1997). The structure of Radical Probabilism. Erkenntnis.

[8] Csiszár I. (1991). Why Least Squares and Maximum Entropy? An Axiomatic Approach to Inference for Linear Inverse Problems. Ann. Stat..

[9] Csiszár I. (1975). I-divergence geometry of probability distributions and minimization problems. Ann. Probab..

[10] Grünwald P.D., Dawid A.P. (2004). Game Theory, Maximum Entropy, Minimum Discrepancy and robust Bayesian Decision Theory. Ann. Stat..

[11] Banerjee A., Guo X., Wang H. (2005). On the Optimality of Conditional Expectation as a Bregman Predictor. IEEE Trans. Inf. Theory.

[12] Frigyik B.A., Srivastava S., Gupta M.R. (2008). Functional Bregman Divergences and Bayesian Estimation of Distributions. IEEE Trans. Inf. Theory.

